# Association between the comprehensive dietary antioxidant index and adult asthma: A cross-sectional study based on NHANES 2003 to 2018

**DOI:** 10.1097/MD.0000000000050226

**Published:** 2026-08-21

**Authors:** Zhizhi Sun, Hanpeng Lai, Rui Sun, Lingfeng Min

**Affiliations:** a The Yangzhou Clinical Medical College, Xuzhou Medical University, Yangzhou, Jiangsu, China; b School of Public Health, Yangzhou University, Yangzhou, Jiangsu, China; c Northern Jiangsu People’s Hospital, Yangzhou University, Yangzhou, Jiangsu, China.

**Keywords:** antioxidants, asthma, cross-sectional studies, diet, nutrition surveys, oxidative stress

## Abstract

Increasing evidence suggests that oxidative stress plays a key role in the pathogenesis of asthma; however, research on the association of the comprehensive dietary antioxidant index (CDAI) with asthma remains limited. Therefore, this study aims to explore the association between CDAI and asthma. Data from the National Health and Nutrition Examination Survey (NHANES) (2003–2018) were analyzed to examine the association between CDAI, derived from two 24-hour dietary recalls, and asthma, defined by self-reported physician diagnosis. Logistic regression was used to examine the overall association, while restricted cubic spline models were applied to explore potential nonlinear relationships. A total of 35,945 adult participants were included, with 14.2% diagnosed with asthma. In the unadjusted model, higher CDAI levels, particularly within a moderate range, were generally associated with a lower risk of asthma. After adjustment for potential confounders, the highest CDAI quartile showed lower odds of asthma than the lowest quartile; however, this comparison did not reach statistical significance (odds ratio = 0.88, 95% confidence interval: 0.76–1.02; *P* = .078). Importantly, restricted cubic spline analysis demonstrated a significant nonlinear association between CDAI and asthma (*P* for nonlinearity <.05), with asthma risk declining at moderate CDAI levels and rising at very high levels. Subgroup analyses showed stronger protective effects among adults aged 40 to 60 years, females, non-Hispanic Blacks, other Hispanic populations, former smokers, and individuals with obesity. Sensitivity analyses indicated that most individual dietary antioxidants exhibited nonlinear associations with asthma, except for vitamin A. CDAI is nonlinearly associated with asthma risk, with moderate levels generally reducing risk, while very high intake may diminish this benefit, highlighting the role of antioxidant-rich diets in asthma prevention. Future prospective cohort studies and mechanistic investigations are warranted to clarify causal relationships and to determine optimal dietary antioxidant intake ranges for asthma prevention.

## 1. Introduction

Asthma is a common chronic respiratory disease characterized by airway inflammation, airway hyperresponsiveness, and reversible airflow obstruction. Clinically, it manifests as recurrent wheezing, shortness of breath, chest tightness, and coughing. According to the global initiative for asthma report, approximately 300 million people worldwide are affected by asthma, with high morbidity and mortality rates, making it a major public health issue.^[1]^ In the United States, asthma affects approximately 25 million individuals, representing nearly 8% of the population. According to data from the Centers for Disease Control and Prevention, asthma is associated with considerable healthcare utilization and economic burden. Although mortality rates have declined in recent years, asthma-related deaths continue to occur annually.^[2]^ Reports from the American Lung Association further underscore the significant morbidity and public health impact of asthma in the U.S. population.^[3]^ Therefore, identifying and intervening in modifiable risk factors is crucial for reducing the disease burden, improving prognosis, and formulating public health strategies.

Oxidative stress plays a critical role in the pathogenesis of asthma and has been shown to aggravate airway inflammation, thereby influencing disease severity.^[4–6]^ Dietary antioxidants may mitigate these effects through several mechanisms. Vitamins C and E directly scavenge reactive oxygen species, reducing oxidative damage to airway epithelial cells. Carotenoids help regulate immune responses and suppress pro-inflammatory cytokine release, while selenium and zinc act as cofactors for antioxidant enzymes such as glutathione peroxidase and superoxide dismutase, maintaining redox balance. Together, these actions may lessen airway hyperresponsiveness, mucus hypersecretion, and structural remodeling. Consistent with these mechanisms, higher dietary antioxidant intake has been associated with reduced risks of chronic diseases, including hypertension, hyperlipidemia, diabetes, and rheumatoid arthritis.^[7–10]^ However, evidence for their protective role in respiratory diseases, particularly asthma, remains limited.

The comprehensive dietary antioxidant index (CDAI) is a validated tool designed to quantify an individual’s overall dietary antioxidant capacity, integrating key antioxidants including vitamins A, C, and E, selenium, zinc, and β-carotenoids.^[11]^ Although oxidative stress is a recognized mechanism in asthma pathogenesis, quantitative evidence linking total dietary antioxidant capacity to asthma remains absent. Leveraging nationally representative data from U.S. adults, this study systematically quantified the association between CDAI and asthma risk, with the aim of clarifying the potential dose–response relationship and informing antioxidant-oriented dietary interventions for asthma prevention.

## 2. Materials and methods

### 2.1. Study population

**Figure 1.**
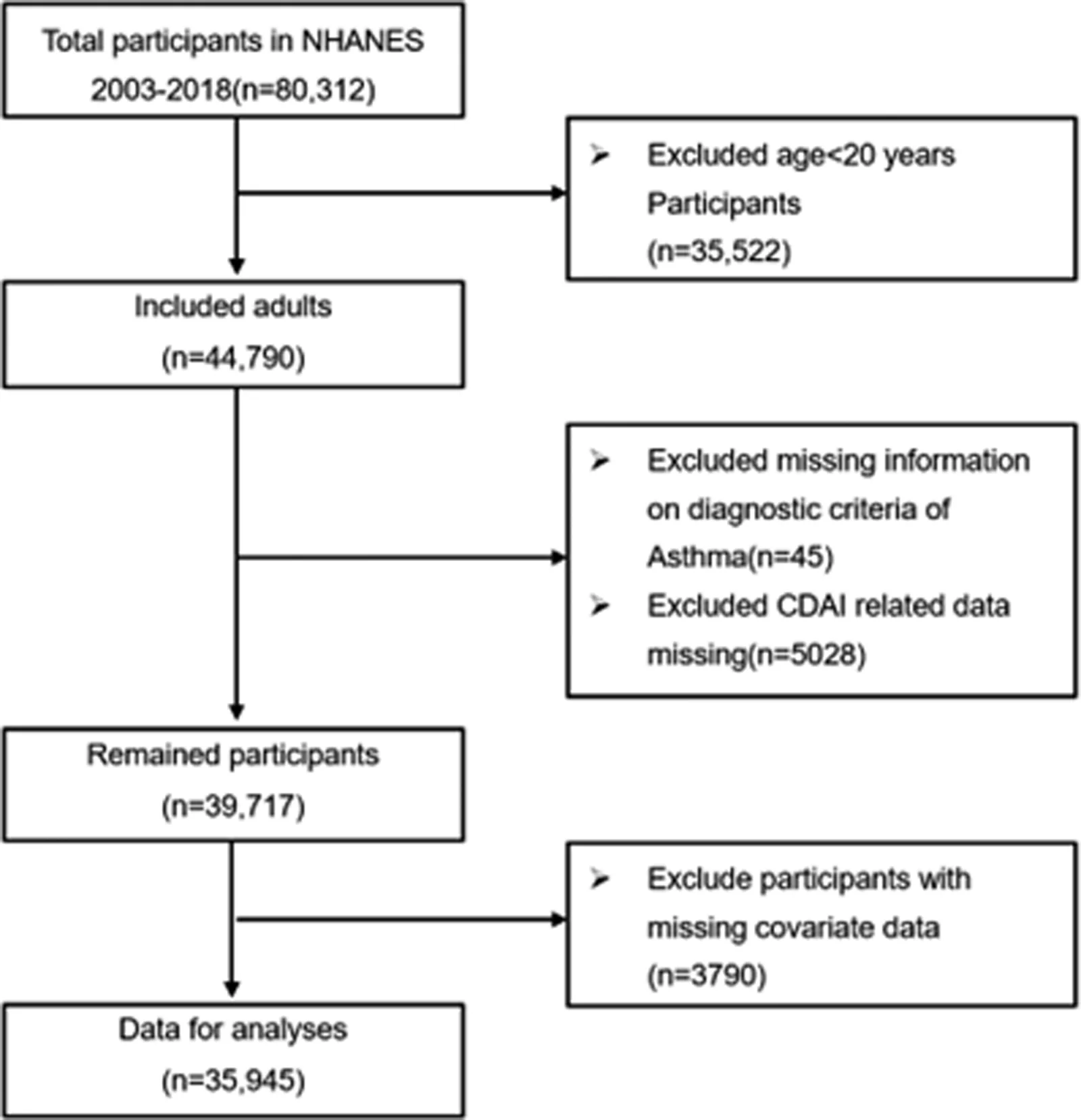
A detailed flow chart of participant recruitment. CDAI = comprehensive dietary antioxidant index, NHANES = National Health and Nutrition Examination Survey.

The National Health and Nutrition Examination Survey (NHANES) is a series of cross-sectional surveys conducted by the National Center for Health Statistics to assess the health and nutritional status of the U.S. population. The survey collects information on demographics, socioeconomic status, dietary intake, and health-related factors through interviews, physical examinations, and laboratory tests. Since 1999, NHANES data have been continuously updated and released in 2-year cycles.^[12]^ The data used in this study were approved by the National Center for Health Statistics Research Ethics Review Board, and all participants provided written informed consent prior to participation. For the present analysis, NHANES data from 2003 to 2018 across 8 consecutive survey cycles were included, comprising 80,312 participants. Individuals under 20 years of age, those with missing information on asthma diagnosis or intake of the 6 dietary antioxidants (vitamins A, C, and E, zinc, selenium, and β-carotene), and participants with missing key covariates were sequentially excluded, resulting in a final sample of 35,945 participants. The participant selection process is illustrated in Figure 1.

### 2.2. Exposure and outcome

In NHANES, dietary antioxidant intake was assessed through two 24-hour dietary recall interviews. The 1st dietary recall was conducted in person by a trained interviewer at the mobile examination center, while the 2nd was conducted by telephone 3 to 10 days later. Averaging intake data from 2 nonconsecutive days is considered more reliable than relying on a single day. From these recalls, intake levels of 6 dietary antioxidants (vitamins A, C, and E, zinc, selenium, and carotenoids) were extracted. Each antioxidant intake was then standardized by subtracting the mean and dividing by the standard deviation, and the CDAI was calculated as the sum of these standardized values.^[13,14]^ Asthma status was obtained from the NHANES questionnaire. Specifically, participants were asked whether a doctor or other health professional had ever diagnosed them with asthma (MCQ010); those who answered “yes” were classified as having asthma.

### 2.3. Covariates

To account for potential confounding factors, the following covariates were included: age, gender, ethnicity, education level, economic status, body mass index (BMI), smoking status, and histories of hypertension and diabetes. Ethnicity was classified as Mexican American, non-Mexican Hispanic, non-Hispanic White, non-Hispanic Black, and other/multiracial. Education level was categorized as below high school and high school and above. BMI was grouped as underweight (≤18.5 kg/m^2^), normal (18.6–24.9 kg/m^2^), overweight (25.0–29.9 kg/m^2^), and obese (≥30 kg/m^2^). Household income was expressed as the poverty-income ratio (PIR) and classified as low (<1.3), middle (1.3–3.5), and high (≥3.5). Smoking status was classified as never (<100 cigarettes lifetime), current (smoking at the time of the survey), or former (≥100 cigarettes lifetime but not currently smoking). Histories of hypertension and diabetes were determined based on self-reported questionnaire responses.

### 2.4. Statistical analysis

Participants were 1st divided into 2 groups based on the presence or absence of asthma and further stratified into 4 subgroups based on CDAI quartiles. Continuous variables were presented as mean ± standard deviation, while categorical variables were expressed as percentages. Between-group comparisons were performed using *t* tests for continuous variables and chi-square or Fisher exact tests for categorical variables. To investigate the association between CDAI and asthma, we constructed multivariable logistic regression models. Model 1 was an unadjusted crude model. Model 2 was adjusted for gender, age, and ethnicity, and model 3 was additionally adjusted for education level, socioeconomic status, BMI, smoking status, hypertension, and diabetes. Restricted cubic spline (RCS) analysis was then employed to assess potential nonlinear associations between CDAI and asthma, followed by stratified analyses. To evaluate the robustness of our findings, sensitivity analyses were also conducted. All statistical analyses were performed using R software (version 4.3.3; R Foundation for Statistical Computing), and a 2-tailed *P* value <.05 was considered statistically significant.

## 3. Results

### 3.1. Baseline characteristics

This study included 35,945 participants, comprising 30,830 non-asthmatic individuals and 5115 asthmatic patients, with a mean age of 49.31 ± 17.94 years. Compared with the non-asthmatic group, asthmatic patients were more likely to be female, younger, non-Hispanic White or non-Hispanic Black, have higher education levels, higher BMI, lower PIR, and a higher prevalence of smoking, hypertension, and diabetes (all *P* < .01). In addition, asthmatic patients had lower CDAI scores than non-asthmatic individuals (*P* < .05). Detailed characteristics are presented in Table 1.

**Table 1:** Baseline characteristics grouped by asthma.

Characteristic	Overall (N = 35,945)	Non-asthma (N = 30,830)	Asthma (N = 5115)	*P* value
Age, yr	49.31 (17.94)	49.68 (17.94)	47.02 (17.78)	<.001
Gender				<.001
Male	17,423 (48%)	15,298 (50%)	2125 (42%)	
Female	18,522 (52%)	15,532 (50%)	2990 (58%)	
Ethnicity				<.001
Non-Hispanic White	16,257 (45%)	13,772 (45%)	2485 (49%)	
Non-Hispanic Black	7613 (21%)	6353 (21%)	1260 (25%)	
Mexican American	5626 (16%)	5144 (17%)	482 (9.4%)	
Other Hispanic	2989 (8.3%)	2543 (8.2%)	446 (8.7%)	
Other/multiracial	3460 (9.6%)	3018 (9.8%)	442 (8.6%)	
Education level				<.001
Below high school	8689 (24%)	7585 (25%)	1104 (22%)	
High school	8365 (23%)	7218 (23%)	1147 (22%)	
Above high school	18,891 (53%)	16,027 (52%)	2864 (56%)	
PIR				<.001
Low	11,081 (31%)	9227 (30%)	1854 (36%)	
Middle	13,724 (38%)	11,930 (39%)	1794 (35%)	
High	11,140 (31%)	9673 (31%)	1467 (29%)	
Smoking status				<.001
Never	19,547 (54%)	17,001 (55%)	2546 (50%)	
Current	7488 (21%)	6227 (20%)	1261 (25%)	
Former	8910 (25%)	7602 (25%)	1308 (26%)	
BMI, kg/m^2^	29.17 (6.94)	28.90 (6.68)	30.75 (8.14)	<.001
Hypertension				<.001
No	23,149 (64%)	20,141 (65%)	3008 (59%)	
Yes	12,796 (36%)	10,689 (35%)	2107 (41%)	
Diabetes				<.001
No	30,721 (85%)	26,517 (86%)	4204 (82%)	
Yes	5224 (15%)	4313 (14%)	911 (18%)	
CDAI	0.66 (4.33)	0.68 (4.28)	0.53 (4.67)	.034

Continuous variables are presented as mean ± standard deviation, and categorical variables are presented as n (%). *P* values were calculated using analysis of variance for continuous variables and chi-square tests for categorical variables.

BMI = body mass index, CDAI = comprehensive dietary antioxidant index, PIR = poverty-income ratio.

### 3.2. The association between CDAI and asthma

Participants were stratified into quartiles according to CDAI levels (*Q*1–*Q*4). Significant differences across quartiles were observed in age, gender, ethnicity, education level, smoking status, socioeconomic status, BMI, and the prevalence of hypertension and diabetes (all *P* < .05). Notably, as CDAI quartiles increased, the prevalence of asthma declined, from 16% in the *Q*1 to 14% in the *Q*4 (*P* < .001). Detailed results are shown in Table 2.

**Table 2:** Basic characteristics of participants by CDAI quartiles.

Characteristic	Overall (N = 35,945)	*Q*1 (N = 8986)	*Q*2 (N = 8986)	*Q*3 (N = 8986)	*Q*4 (N = 8987)	P value
Age, yr	49.31 (17.94)	50.25 (18.35)	50.04 (18.16)	49.11 (17.82)	47.82 (17.30)	<.001
Gender						<.001
Male	17,423 (48%)	3162 (35%)	3935 (44%)	4628 (52%)	5698 (63%)	
Female	18,522 (52%)	5824 (65%)	5051 (56%)	4358 (48%)	3289 (37%)	
Ethnicity						<.001
Non-Hispanic White	16,257 (45%)	3694 (41%)	4049 (45%)	4250 (47%)	4264 (47%)	
Non-Hispanic Black	7613 (21%)	2318 (26%)	1878 (21%)	1699 (19%)	1718 (19%)	
Mexican American	5626 (16%)	1417 (16%)	1502 (17%)	1416 (16%)	1291 (14%)	
Other Hispanic	2989 (8.3%)	843 (9.4%)	762 (8.5%)	712 (7.9%)	672 (7.5%)	
Other/multiracial	3460 (9.6%)	714 (7.9%)	795 (8.8%)	909 (10%)	1042 (12%)	
Education level						<.001
Below high school	8689 (24%)	2959 (33%)	2291 (25%)	1888 (21%)	1551 (17%)	
High school	8365 (23%)	2369 (26%)	2155 (24%)	1992 (22%)	1849 (21%)	
Above high school	18,891 (53%)	3658 (41%)	4540 (51%)	5106 (57%)	5587 (62%)	
PIR						<.001
Low	11,081 (31%)	3592 (40%)	2850 (32%)	2372 (26%)	2267 (25%)	
Middle	13,724 (38%)	3523 (39%)	3510 (39%)	3485 (39%)	3206 (36%)	
High	11,140 (31%)	1871 (21%)	2626 (29%)	3129 (35%)	3514 (39%)	
Smoking status						<.001
Never	19,547 (54%)	4582 (51%)	4951 (55%)	4980 (55%)	5034 (56%)	
Current	7488 (21%)	2471 (27%)	1808 (20%)	1626 (18%)	1583 (18%)	
Former	8910 (25%)	1933 (22%)	2227 (25%)	2380 (26%)	2370 (26%)	
BMI, kg/m^2^	29.17 (6.94)	29.43 (7.18)	29.45 (6.84)	29.20 (6.98)	28.58 (6.71)	<.001
Hypertension						<.001
No	23,149 (64%)	5470 (61%)	5642 (63%)	5969 (66%)	6068 (68%)	
Yes	12,796 (36%)	3516 (39%)	3344 (37%)	3017 (34%)	2919 (32%)	
Diabetes						<.001
No	30,721 (85%)	7526 (84%)	7570 (84%)	7766 (86%)	7859 (87%)	
Yes	5224 (15%)	1460 (16%)	1416 (16%)	1220 (14%)	1128 (13%)	
Asthma						<.001
No	30,830 (86%)	7551 (84%)	7723 (86%)	7825 (87%)	7731 (86%)	
Yes	5115 (14%)	1435 (16%)	1263 (14%)	1161 (13%)	1256 (14%)	

Continuous variables are presented as mean ± standard deviation, and categorical variables are presented as n (%). *P* values were calculated using analysis of variance for continuous variables and chi-square tests for categorical variables.

BMI = body mass index, CDAI = comprehensive dietary antioxidant index, PIR = poverty-income ratio.

Building on these descriptive findings, logistic regression models were constructed to further assess the association between CDAI and asthma risk by adjusting for covariates including age, gender, ethnicity, education level, socioeconomic status, BMI, smoking status, hypertension, and diabetes. Using the lowest CDAI quartile (*Q*1) as the reference, a significant inverse association between CDAI levels and asthma risk was observed (*P* < .05). Notably, in all models, the effect decreased from *Q*2 to *Q*3 but increased from *Q*3 to *Q*4, indicating a non-monotonic trend. In model 3, the association between the highest CDAI quartile (*Q*4) and asthma risk did not reach statistical significance (odds ratio = 0.88, 95% confidence interval: 0.76–1.02, *P* = .078). Trend tests indicated that asthma risk consistently decreased with increasing CDAI levels across all models (*P* for trend <.001) (Table 3).

**Table 3:** Association between CDAI and asthma.

CDAI	Model 1		Model 2		Model 3	
	OR (95% CI)	*P*	OR (95% CI)	*P*	OR (95% CI)	*P*
*Q*1	Ref	Ref	Ref	Ref	Ref	Ref
*Q*2	0.85 (0.78–0.92)	<.001	0.87 (0.80–0.95)	.002	0.88 (0.81–0.97)	.007
*Q*3	0.76 (0.68–0.84)	<.001	0.79 (0.71–0.87)	<.001	0.81 (0.73–0.90)	<.001
*Q*4	0.80 (0.69–0.92)	.002	0.84 (0.73–0.98)	.021	0.88 (0.76–1.02)	.078
*P* for trend		<.001		<.001		<.001

Model 1 was unadjusted. Model 2 was adjusted for age, gender, and ethnicity. Model 3 was further adjusted for education level, poverty-income ratio, smoking status, body mass index, hypertension, and diabetes.

CDAI = comprehensive dietary antioxidant index, CI = confidence interval, OR = odds ratio, Ref = reference category.

**Figure 2.**
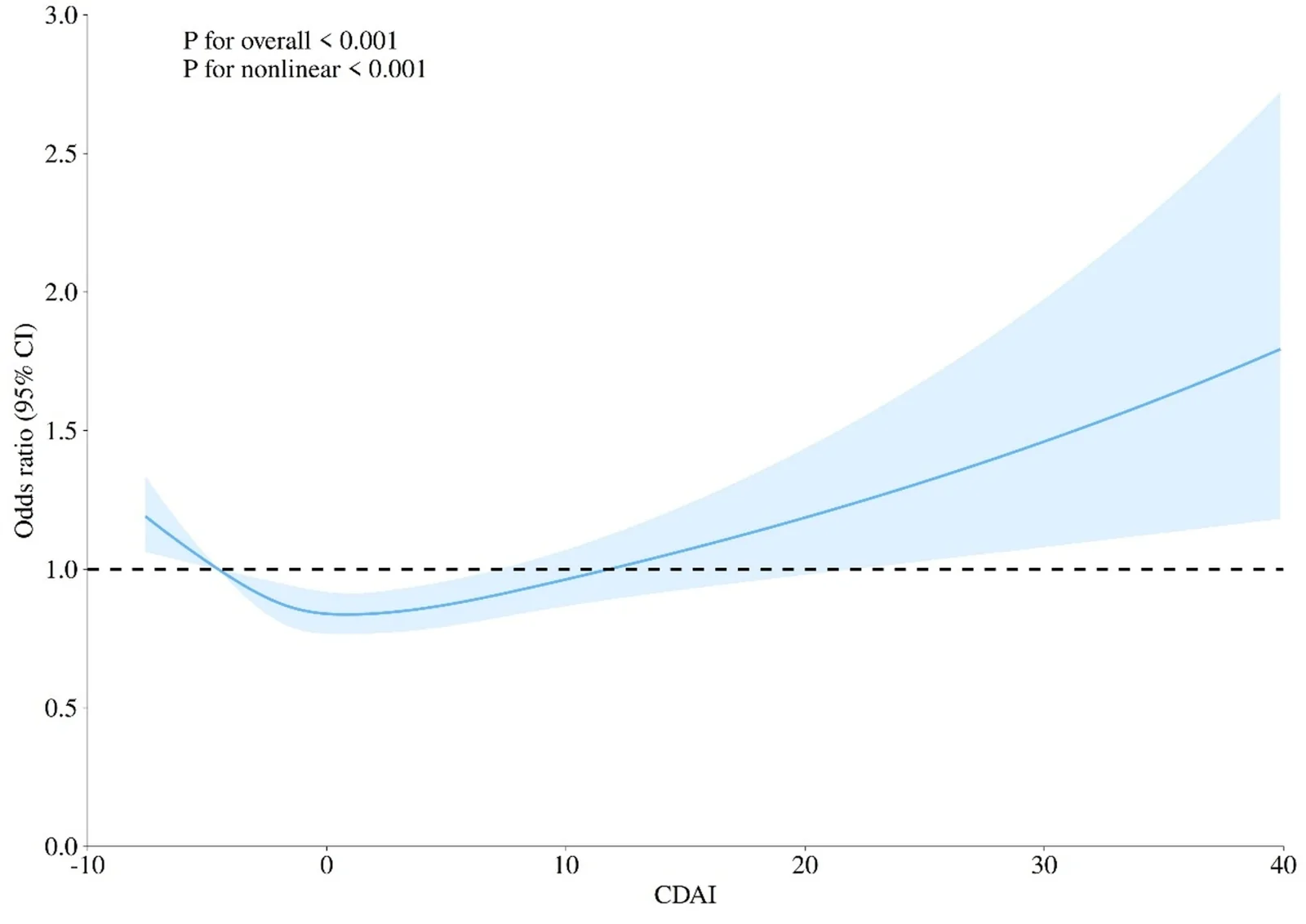
RCS for the relationship between CDAI and asthma. CDAI = comprehensive dietary antioxidant index, CI = confidence interval, RCS = restricted cubic spline.

To further verify this non-monotonic relationship, RCS analysis was conducted, revealing a significant nonlinear association between CDAI and asthma risk (*P* for nonlinearity <.05), with asthma risk declining as CDAI increased up to 0 and rising thereafter (Fig. 2).

### 3.3. Subgroup analysis

**Figure 3.**
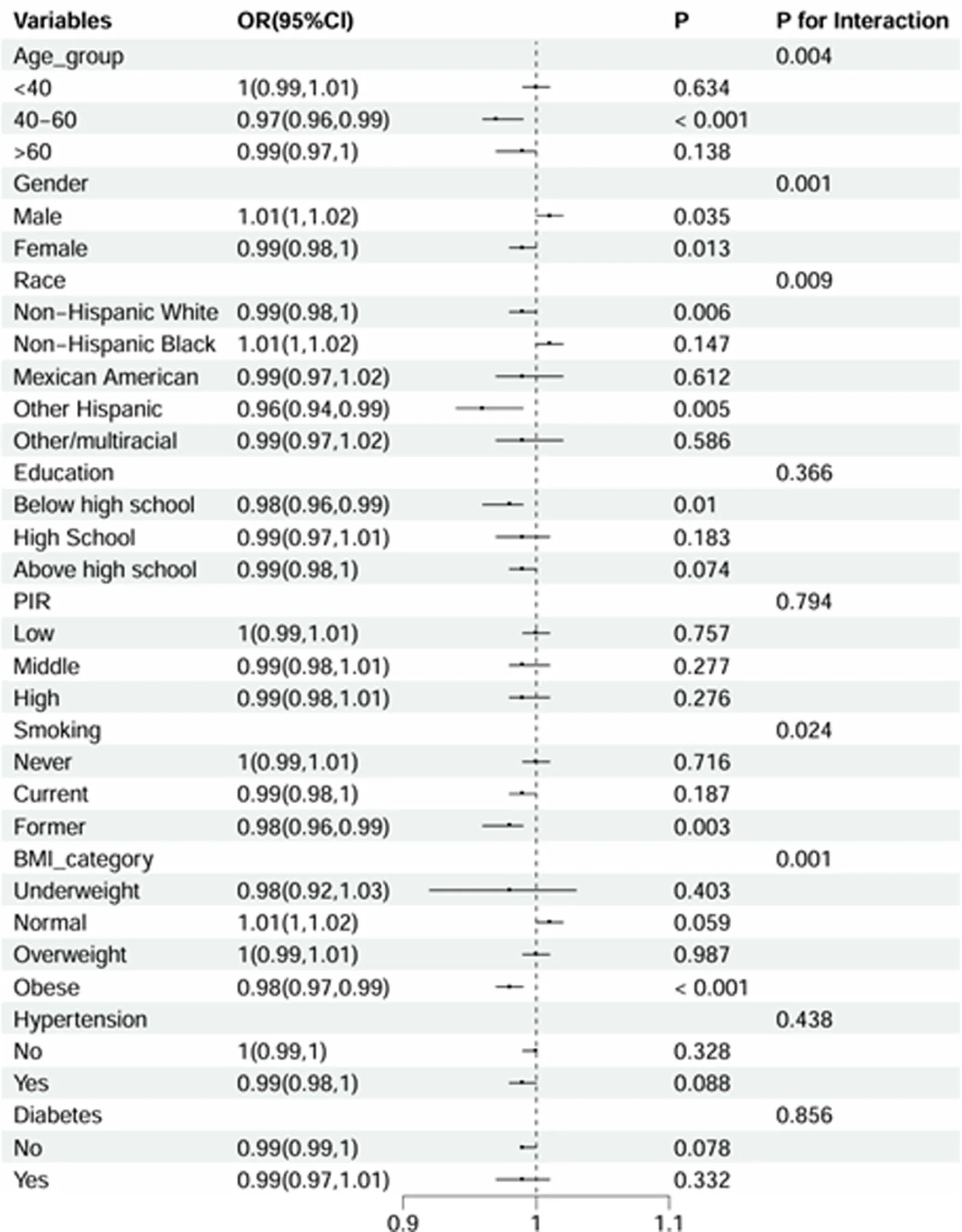
Subgroup analysis for the relationship between CDAI and asthma. BMI = body mass index, CI = confidence interval, OR = odds ratio, PIR = poverty-income ratio.

Subgroup analyses were conducted to assess the association between CDAI and asthma risk in specific populations. After adjustment for confounding factors, no significant interactions were detected in most subgroups, with the exception of age, gender, ethnicity, smoking status, and BMI (all *P* > .05). Further analysis indicated that higher CDAI levels were significantly associated with a reduced risk of asthma among participants aged 40 to 60 years, females, non-Hispanic Blacks, other Hispanic populations, former smokers, and individuals with obesity (Fig. 3).

### 3.4. Sensitivity analysis

**Figure 4.**
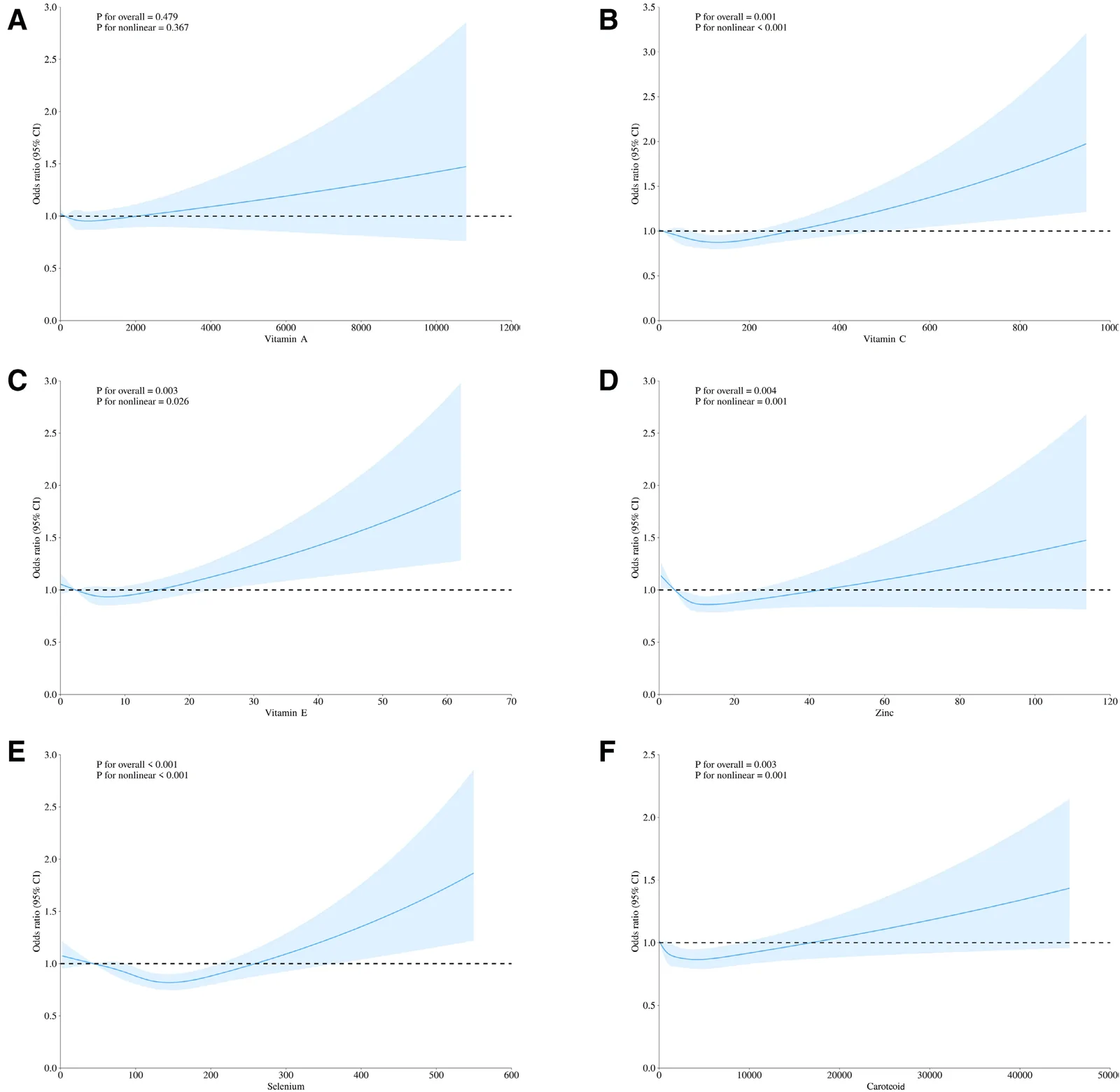
RCS for the relationships between single antioxidants and asthma. (A) Vitamin A, (B) Vitamin C, (C) Vitamin E, (D) Zinc, (E) Selenium, (F) Carotenoid. CI = confidence interval, RCS = restricted cubic spline

To evaluate the robustness of the findings, we further examined the associations between individual dietary antioxidants and asthma. As shown in Figure 4, after adjustment for all potential confounders, significant nonlinear relationships with asthma risk persisted for most dietary antioxidants, with the exception of vitamin A (*P* for trend <.05).

## 4. Discussion

This study, based on NHANES data, conducted a cross-sectional analysis to examine the association between the CDAI and asthma. The findings suggest that moderate increases in CDAI are associated with a reduced risk of asthma, implying that CDAI may act as a potential protective factor within an optimal range of intake. This association remained robust after adjusting for confounding factors, including age, gender, ethnicity, education level, PIR, BMI, smoking status, hypertension, and diabetes. Moreover, the RCS analysis revealed a significant nonlinear relationship between CDAI and asthma. Subgroup analysis further demonstrated that the protective effect of CDAI was particularly evident among individuals aged 40 to 60 years, females, other Hispanic populations, former smokers, and those with obesity. Sensitivity analysis also showed that dietary intake of vitamin C, vitamin E, zinc, selenium, and carotenoids exhibited nonlinear associations with asthma risk.

Asthma is one of the most common chronic diseases worldwide, yet its pathogenesis remains incompletely understood. Currently, it is thought to involve airway immune-inflammatory mechanisms, neuroregulatory mechanisms, and their interactions.^[15]^ In addition, numerous studies have demonstrated a close link between oxidative stress and asthma. The onset of asthma can elevate oxidative stress levels, with excessive reactive oxygen species directly damaging airway epithelial cells and interacting with chronic airway inflammation, thereby creating a vicious cycle that promotes disease progression.^[16–19]^ Increasing evidence also indicates that diet, as an external factor, plays a critical role in regulating oxidative stress levels in the body.^[20–22]^

The 6 dietary antioxidants examined in this study, vitamins A, C, and E, zinc, selenium, and carotenoids, are widely recognized for their antioxidant properties and regulatory effects on oxidative stress.^[23–25]^ The CDAI, which quantifies overall dietary antioxidant intake, provides a more holistic assessment of diet quality compared to evaluating individual antioxidants alone. Consequently, it has been increasingly applied in clinical research. A growing body of evidence indicates that CDAI is associated with the risk of various diseases,^[26–28]^ including respiratory conditions. Previous studies investigating the association between CDAI and asthma have typically focused on specific age groups, such as children and adolescents (1–18 years) or adults over 20 years old, and often relied on different datasets, yielding inconsistent findings. In contrast, the present study utilizes nationally representative NHANES data, encompassing a broad adult age range (20 years and older), thereby providing a more comprehensive and robust assessment of the relationship between CDAI and asthma.^[29–31]^

In the present study, focusing on U.S. adults aged 20 years and older, we found that after adjusting for potential confounders, higher CDAI levels were significantly associated with lower odds ratios for asthma, suggesting a potential protective role of CDAI. However, at the highest CDAI levels, the protective effect appeared attenuated, indicating that maintaining CDAI within an optimal range may be most beneficial. Indeed, excessive antioxidant intake is not necessarily advantageous; oversupplementation has been linked to increased risk of certain diseases, such as lung cancer and lipid abnormalities.^[32–34]^ Since CDAI reflects the combined intake of 6 antioxidants, we hypothesize that interactions among these components may influence asthma risk, such that higher CDAI levels do not always correspond to proportionally lower risk.

Furthermore, the nonlinear, non-monotonic relationship between CDAI and asthma was confirmed by RCS analysis. Asthma risk was lowest when CDAI was around 0, but increased at values above 0. This pattern may reflect that moderate antioxidant intake optimizes the balance between reactive oxygen species and antioxidant defenses, whereas excessive intake may disrupt this balance, impair physiological oxidative signaling, or even exert pro-oxidant effects. Moreover, interactions among the 6 antioxidants may produce diminishing or counteractive effects at higher intake levels, resulting in the observed non-monotonic association between CDAI and asthma risk. Further evidence from other sources is needed to explore in depth the observed attenuation of the protective effect at the highest CDAI levels, in order to elucidate the underlying mechanisms and to determine an appropriate recommended intake range.

Interestingly, vitamin A exhibited a linear positive association with asthma risk, in contrast to the nonlinear patterns observed for the other 5 antioxidants. This may be explained by its tightly regulated physiological window: serum and tissue levels of vitamin A are strictly controlled, and intake beyond the optimal range can quickly reach potentially harmful levels. Excessive vitamin A has been associated with hepatotoxicity and pro-inflammatory effects, which may directly impact respiratory health and increase asthma risk. Moreover, unlike other antioxidants that may interact or buffer each other’s effects, vitamin A primarily acts independently, lacking compensatory interactions. Consequently, even modest increases above the optimal level may rapidly elevate asthma risk, resulting in the observed linear relationship. From a public health perspective, the linear increase in asthma risk with higher vitamin A intake underscores caution in supplementation, suggesting that even moderate increases may be potentially harmful. Public health strategies should prioritize avoiding excessive vitamin A intake rather than aiming for a specific target level.

Compared with the lowest CDAI quartile, higher quartiles were associated with a lower proportion of asthma cases, suggesting that while low CDAI intake may be detrimental, moderate levels may be optimal for health. To examine whether this association differed across specific population subgroups, given that effects may vary by age, sex, race, smoking status, or obesity, we performed subgroup analyses. Before adjustment, the inverse correlation between CDAI and asthma was observed across all subgroups. After adjusting for confounding factors, this significant negative correlation remained particularly evident among adults aged 40 to 60 years, females, non-Hispanic Blacks, other Hispanic populations, former smokers, and individuals with obesity, possibly reflecting differences in physiology, lifestyle, and environmental exposures that modulate the protective effect of dietary antioxidants. In our baseline comparisons, participants with asthma tended to be younger in this NHANES analytic sample. This pattern may differ from age-specific estimates in other U.S. surveillance reports and may reflect differences in survey design, weighting, and asthma definitions. Importantly, this descriptive age distribution is distinct from our subgroup finding that the inverse CDAI-asthma association was more pronounced among adults aged 40 to 60 years, suggesting potential effect modification by age. These findings suggest that dietary interventions aimed at increasing antioxidant intake could be particularly beneficial for these specific subgroups. Public health strategies or nutrition programs may consider prioritizing middle-aged adults, females, certain racial/ethnic populations, former smokers, and individuals with obesity when designing targeted antioxidant-rich dietary recommendations to reduce asthma risk.

This study has several strengths and limitations. A primary strength is the use of 16 years of continuous data from a public database, with a large sample size that enhances the stability and reliability of the findings. Additionally, we thoroughly investigated the nonlinear relationship between CDAI and adult asthma, providing valuable evidence to inform reasonable dietary intake. However, several limitations should be acknowledged. First, dietary intake was assessed using 2 nonconsecutive 24-hour dietary recalls. Although averaging 2 recalls improves reliability compared with a single-day assessment, previous literature suggests that 3-day or repeated dietary assessments may provide more accurate estimates of habitual intake, particularly for nutrients with high day-to-day variability, such as vitamins and antioxidants. Therefore, measurement error and within-person variability may have influenced the estimation of CDAI. Second, asthma status was determined based on self-reported physician diagnosis rather than objective spirometry-based criteria. Although spirometry is considered the gold standard for diagnosing obstructive airway diseases, such measurements were not uniformly available for all NHANES participants included in this analysis. Consequently, misclassification bias may exist. In addition, the questionnaire did not distinguish between current active asthma and historical diagnosis, which may further contribute to outcome misclassification. Third, physical activity was not included as a covariate in the present analysis. Given that physical activity is associated with both dietary quality and respiratory health, residual confounding cannot be entirely excluded. Finally, as this was a cross-sectional study, causal relationships between CDAI and adult asthma cannot be established. Prospective cohort studies with larger sample sizes are warranted to confirm these findings, clarify temporal relationships, and further refine the potential threshold effect.

## 5. Conclusions

This study concludes that CDAI is nonlinearly associated with asthma risk in a population-specific manner. Relatively higher CDAI levels are generally linked to reduced asthma risk, whereas very high intake, particularly of vitamin A, may reverse this benefit. Similar patterns were observed in adults aged 40 to 60 years, females, non-Hispanic Blacks, other Hispanic populations, former smokers, and individuals with obesity. These findings suggest that optimizing, rather than maximizing, dietary antioxidant intake may be important for asthma prevention.

## Acknowledgments

The authors express their sincere gratitude to the participants and team members of NHANES.

## Author contributions

**Conceptualization:** Zhizhi Sun.

**Data curation:** Zhizhi Sun, Rui Sun.

**Formal analysis:** Zhizhi Sun.

**Funding acquisition:** Lingfeng Min.

**Methodology:** Zhizhi Sun.

**Project administration:** Lingfeng Min.

**Resources:** Lingfeng Min.

**Software:** Zhizhi Sun.

**Supervision:** Hanpeng Lai, Lingfeng Min.

**Validation:** Zhizhi Sun, Rui Sun.

**Writing – original draft:** Zhizhi Sun.

**Writing – review & editing:** Hanpeng Lai, Lingfeng Min.
